# Supplementary material for: Effectiveness of ChAdOx1-S COVID-19 booster vaccination against the Omicron and Delta variants in England
Source: Nat Commun. 2022 Dec 12;13:7688. doi: 10.1038/s41467-022-35168-7 (PMC9744366; doi:10.1038/s41467-022-35168-7)
Supplement: Supplementary file 1 — Supplementary Information [file 41467_2022_35168_MOESM1_ESM.pdf]

Supplementary Information

Supplementary Tables

Supplementary Table 1. SUS Acute respiratory illness ICD10 code list.

SUS Acute respiratory illness ICD10 code list	
J04*	Acute laryngitis and tracheitis
J09*	Influenza due to identified avian influenza virus
J10*	Influenza with pneumonia, other influenza virus identified
J11*	Influenza with pneumonia, virus not identified
J12*	Viral pneumonia, not elsewhere classified
J13*	Pneumonia due to Streptococcus pneumoniae
J14*	Pneumonia due to Haemophilus influenzae
J15*	Bacterial pneumonia, not elsewhere classified
J16*	Pneumonia due to other infectious organisms, not elsewhere classified
J17*	Pneumonia in diseases classified elsewhere
J18*	Pneumonia, organism unspecified
J20*	Acute bronchitis
J21*	Acute bronchiolitis
J22*	Unspecified acute lower respiratory infection
J80*	ARDS (related to respiratory infection)
U07*	COVID-19, virus identified and not identified
U04*	Severe acute respiratory syndrome (SARS)

Supplementary Table 2. Investigated outcomes - definitions and data sources

Outcome	Definition and Data sources
Symptomatic disease	Table 3, Supplementary Table 4. Cases were identified from community testing data only (Pillar 2) and were restricted to persons who had reported symptoms and gave a symptom onset date within the 10 days before testing. These VE estimates reflect protection against mild disease.
Hospitalisation (primary analysis)	Figure 1, Table 4. The primary hospitalisation analysis estimated VE using Pillar 1 and Pillar 2 tests which could be linked to the SUS hospitalisation records. Pillar 2 tests were identified where the sample was taken 14 days before and up to 2 days after the day of admission. Pillar 1 tests were included where the sample was taken 1 day before and up to 2 days after the admission. Only tests linked to an admission with a respiratory code in the primary diagnosis field and where the length of stay was at least 2 days were included. These VE estimates reflect protection against hospitalisation using a strict definition to limit contamination with incidental admissions.
Hospitalisation (secondary analysis)	Supplementary Table 5. The secondary hospitalisation analysis estimated VE in a subset of Pillar 2 community tests from symptomatic individuals; those for which an admission to accident and emergency (ECDS) occurred in the 14 days after their test date. These VE estimates reflect protection against hospitalisation using a broader definition which may include contamination with incidental admissions and therefore be more reflective of VE against mild disease.

Supplementary Table 3. Descriptive characteristics of those included in the VE analysis for protection against symptomatic disease (cases all) and hospitalisation as assessed using the ECDS data following infection with either Delta or Omicron variants.

				Delta						Omicron					
				Controls		Cases All		Cases Hosp		Controls		Cases All		Cases Hosp	
	Dose	Booster manufacturer	Interval (days)	n	%	n	%	n	%	n	%	n	%	n	%
				469,976		172,223		1,352		457,377		434,514		509	
Vaccination Status	Unvaccinated			49,918	10.6%	48,699	28.3%	366	27.1%	29,062	6.4%	53,626	12.3%	93	18.3%
	Dose 2*		175+	142,454	30.3%	92,917	54.0%	809	59.8%	89,641	19.6%	92,283	21.2%	84	16.5%
	Booster	Any	0-6	51,109	10.9%	21,257	12.3%	96	7.1%	40,877	8.9%	29,617	6.8%	5	1.0%
	Booster	BNT162b2	7+	225,962	48.1%	9,303	5.4%	76	5.6%	297,056	64.9%	258,297	59.4%	320	62.9%
	Booster	ChAdOx1-S	7+	533	0.1%	47	0.0%	5	0.4%	741	0.2%	691	0.2%	7	1.4%
Gender	Female			284,136	60.5%	91,669	53.2%	624	46.2%	274,565	60.0%	230,789	53.1%	250	49.1%
	Male			185,025	39.4%	80,291	46.6%	728	53.8%	182,025	39.8%	203,031	46.7%	258	50.7%
	Missing			815	0.2%	263	0.2%	0	0.0%	787	0.2%	694	0.2%	1	0.2%
Age	40-44			80,921	17.2%	35,639	20.7%	0	0.0%	83,475	18.3%	94,518	21.8%	0	0.0%
	45-49			74,782	15.9%	30,300	17.6%	0	0.0%	78,924	17.3%	86,768	20.0%	0	0.0%
	50-54			89,098	19.0%	32,587	18.9%	0	0.0%	90,779	19.8%	87,744	20.2%	0	0.0%
	55-59			76,254	16.2%	25,591	14.9%	0	0.0%	75,520	16.5%	67,858	15.6%	0	0.0%
	60-64			55,023	11.7%	18,038	10.5%	0	0.0%	52,087	11.4%	41,777	9.6%	0	0.0%
	65-69			40,628	8.6%	12,894	7.5%	405	30.0%	34,908	7.6%	24,659	5.7%	116	22.8%
	70-74			30,900	6.6%	10,104	5.9%	405	30.0%	24,787	5.4%	18,125	4.2%	134	26.3%
	75-79			14,709	3.1%	4,876	2.8%	275	20.3%	11,152	2.4%	8,756	2.0%	103	20.2%
	80-84			4,094	0.9%	1,269	0.7%	144	10.7%	3,064	0.7%	2,332	0.5%	64	12.6%
	85-89			1,999	0.4%	580	0.3%	81	6.0%	1,518	0.3%	1,107	0.3%	49	9.6%
	90+			1,568	0.3%	345	0.2%	42	3.1%	1,163	0.3%	870	0.2%	43	8.4%
Ethnicity	African			3,922	0.8%	1,291	0.7%	14	1.0%	3,701	0.8%	5,492	1.3%	5	1.0%
	Any other Asian background			5,405	1.2%	1,668	1.0%	14	1.0%	5,437	1.2%	6,253	1.4%	6	1.2%
	Any other Black background			1,810	0.4%	787	0.5%	4	0.3%	1,590	0.3%	2,617	0.6%	0	0.0%

	Any other White background	30,310	6.4%	16,093	9.3%	89	6.6%	29,473	6.4%	39,506	9.1%	29	5.7%
	Any other ethnic group	6,239	1.3%	2,579	1.5%	12	0.9%	6,242	1.4%	7,630	1.8%	10	2.0%
	Any other mixed background	1,945	0.4%	774	0.4%	7	0.5%	1,894	0.4%	2,038	0.5%	2	0.4%
	Bangladeshi or British Bangladeshi	1,743	0.4%	678	0.4%	9	0.7%	1,741	0.4%	2,198	0.5%	4	0.8%
	British, Mixed British	355,004	75.5%	123,459	71.7%	1,036	76.6%	345,349	75.5%	302,173	69.5%	383	75.2%
	Caribbean	2,847	0.6%	1,443	0.8%	16	1.2%	2,474	0.5%	4,463	1.0%	9	1.8%
	Chinese	1,451	0.3%	490	0.3%	7	0.5%	1,503	0.3%	1,453	0.3%	3	0.6%
	Indian or British Indian	12,668	2.7%	3,191	1.9%	29	2.1%	12,322	2.7%	11,073	2.5%	7	1.4%
	Irish	3,313	0.7%	921	0.5%	7	0.5%	3,170	0.7%	2,649	0.6%	10	2.0%
	Pakistani or British Pakistani	5,791	1.2%	2,145	1.2%	19	1.4%	5,770	1.3%	7,135	1.6%	10	2.0%
	White and Asian	832	0.2%	301	0.2%	0	0.0%	865	0.2%	834	0.2%	0	0.0%
	White and Black African	656	0.1%	255	0.1%	0	0.0%	655	0.1%	850	0.2%	1	0.2%
	White and Black Caribbean	915	0.2%	404	0.2%	4	0.3%	840	0.2%	1,284	0.3%	2	0.4%
	Missing	35,125	7.5%	15,744	9.1%	85	6.3%	34,351	7.5%	36,866	8.5%	28	5.5%
NHS Region	East of England	55,371	11.8%	19,965	11.6%	127	9.4%	54,106	11.8%	44,943	10.3%	46	9.0%
	London	56,857	12.1%	18,455	10.7%	121	8.9%	55,327	12.1%	61,545	14.2%	59	11.6%
	Midlands	89,571	19.1%	33,599	19.5%	274	20.3%	87,072	19.0%	82,976	19.1%	100	19.6%
	North East	70,243	14.9%	27,797	16.1%	287	21.2%	66,238	14.5%	69,399	16.0%	98	19.3%
	North West	65,199	13.9%	23,730	13.8%	180	13.3%	60,400	13.2%	71,916	16.6%	101	19.8%
	South East	82,624	17.6%	28,623	16.6%	210	15.5%	85,855	18.8%	70,342	16.2%	69	13.6%
	South West	50,110	10.7%	20,054	11.6%	153	11.3%	48,378	10.6%	33,393	7.7%	36	7.1%
	Missing	1	0.0%	0	0.0%	0	0.0%	1	0.0%	0	0.0%	0	0.0%
IMD Quintiles	1	72,363	15.4%	28,710	16.7%	284	21.0%	68,334	14.9%	81,965	18.9%	117	23.0%
	2	84,074	17.9%	32,208	18.7%	275	20.3%	80,641	17.6%	84,654	19.5%	109	21.4%
	3	96,109	20.4%	35,481	20.6%	279	20.6%	93,614	20.5%	85,615	19.7%	102	20.0%
	4	104,605	22.3%	37,662	21.9%	278	20.6%	102,289	22.4%	90,030	20.7%	101	19.8%
	5	111,956	23.8%	37,790	21.9%	232	15.5%	111,706	24.4%	91,371	21.0%	80	13.6%
	Missing	869	0.2%	372	0.2%	4	0.3%	793	0.2%	879	0.2%	0	0.0%

Previously positive	No	410,326	87.3%	169,930	98.7%	1,347	99.6%	391,382	85.6%	395,115	90.9%	490	96.3%
	Yes	59,650	12.7%	2,293	1.3%	5	0.4%	65,995	14.4%	39,399	9.1%	19	3.7%
Vaccine priority groups	Healthcare worker	18,031	3.8%	4,068	2.4%	4	0.3%	14,693	3.2%	11,743	2.7%	1	0.2%
	CEV**	44,973	9.6%	13,147	7.6%	475	35.1%	38,131	8.3%	32,486	7.5%	280	55.0%
	At risk***	119,372	25.4%	40,186	23.3%	14	1.0%	111,789	24.4%	95,944	22.1%	3	0.6%
	Severely immunosuppressed	5,571	1.2%	1,871	1.1%	116	8.6%	4,994	1.1%	5,734	1.3%	83	16.3%

* ChAdOx1-S primary course
** CEV: clinically extremely vulnerable
***At risk is only those under 65

Supplementary Table 4. Effectiveness of the ChAdOx1-S and BNT162b2 booster vaccines against symptomatic disease following infection with the Delta variant for adults aged 40 years and older in England.

Dose	Booster Manufacturer	Interval (days)	Controls	Cases	OR*	VE** (95% CI)
			Age 40-64			
Unvaccinated			47,233	46,399	Baseline	Baseline
Dose 2***		175+	121,274	73,031	0.58 (0.57-0.60)	41.7 (40.5 to 42.8)
Booster	Any****	0-6	45,607	16,762	0.48 (0.47-0.50)	51.5 (50.2 to 52.8)
	BNT162b2	7+	161,582	5,940	0.08 (0.08-0.08)	91.9 (91.6 to 92.1)
	ChAdOx1-S	7+	382	23	0.15 (0.09-0.23)	85.4 (77.4 to 90.6)
			Age 65+			
Unvaccinated			2,685	2,300	Baseline	Baseline
Dose 2***		175+	21,180	19,886	0.67 (0.63-0.72)	32.8 (28.0 to 37.3)
Booster	Any****	0-6	5,502	4,495	0.61 (0.57-0.66)	38.7 (33.7 to 43.3)
	BNT162b2	7+	64,380	3,363	0.1 (0.09-0.11)	90.2 (89.4 to 90.9)
	ChAdOx1-S	7+	151	24	0.44 (0.27-0.70)	56.3 (30.0 to 72.7)

*Odds ratio

**Vaccine effectiveness

***ChAdOx1-S primary course

****ChAdOx1-S or BNT162b2

Supplementary Table 5. Effectiveness of the ChAdOx1-S and BNT162b2 booster vaccines against hospitalisation as defined by linkage to the accident and emergency (ECDS) data following infection with Delta or Omicron variants in adults aged 65 years and older in England.

Dose	Booster Manufacturer	Interval (days)	Controls	Cases	OR*	VE** (95% CI)
			Delta			
Unvaccinated			2,685	366	Baseline	Baseline
Dose 2***		175+	21180	809	0.17 (0.15-0.2)	82.8 (79.9 to 85.3)
Booster	Any****	0-6	5,502	96	0.09 (0.07-0.12)	91.0 (88.5 to 93.0)
	BNT162b2	7+	64380	76	0.01 (0.01-0.02)	98.8 (98.4 to 99.1)
	ChAdOx1-S	7+	151	5	0.35 (0.13-0.91)	65.3 (8.7 to 86.8)
			Omicron			
Unvaccinated			1,561	93	Baseline	Baseline
Dose 2***		175+	4329	84	0.45 (0.32-0.63)	55.4 (37.2 to 68.4)
Booster	Any****	0-6	1,567	5	0.15 (0.06-0.38)	85.2 (61.7 to 94.3)
	BNT162b2	7+	63803	320	0.08 (0.06-0.1)	92.3 (89.8 to 94.2)
	ChAdOx1-S	7+	173	7	0.34 (0.15-0.8)	65.9 (19.9 to 85.4)

*Odds ratio

**Vaccine effectiveness

***ChAdOx1-S primary course

****ChAdOx1-S or BNT162b2

Supplementary Table 6. Descriptive characteristics of those included in the VE analysis for protection against hospitalisation as assessed using the SUS data following infection with either Delta or Omicron variants.

				Delta						Omicron					
				Overall		Controls		Cases		Overall		Controls		Cases	
	Dose	Booster manufacturer	Interval (days)	n	%	n	%	n	%	n	%	n	%	n	%
				23,227	100.0%	17,377	74.8%	5,850	25.2%	11,052	100.0%	9,021	81.6%	2,031	18.4%
Vaccination Status	Unvaccinated			3,327	14.3%	1,327	7.6%	2,000	34.2%	1,120	10.1%	517	5.7%	603	29.7%
	Dose 2*		175+	10,248	44.1%	7,000	40.3%	3,248	55.52%	2,026	18.3%	1,596	17.7%	430	21.2%
	Booster	Any	0-6	1,366	5.9%	1,123	6.5%	243	4.15%	388	3.5%	361	4.0%	27	1.3%
	Booster	BNT162b2	7+	8,239	35.5%	7,884	45.4%	355	6.07%	7,448	67.4%	6,495	72.0%	953	46.9%
	Booster	ChAdOx1-S	7+	47	0.2%	43	0.2%	4	0.07%	70	0.6%	52	0.6%	18	0.9%
Pillar	Pillar 1			20,960	90.2%	16,976	97.7%	3,984	68.1%	10,374	93.9%	8,810	97.7%	1,564	77.0%
	Pillar 2			2,267	9.8%	401	2.3%	1,866	31.9%	678	6.1%	211	2.3%	467	23.0%
Gender	Female			12,333	53.1%	9,594	55.2%	2,739	46.8%	5,955	53.9%	4,940	54.8%	1,015	50.0%
	Male			10,806	46.5%	7,699	44.3%	3,107	53.1%	5,041	45.6%	4,028	44.7%	1,013	49.9%
	Missing			88	0.4%	84	0.5%	4	0.1%	56	0.5%	53	0.6%	3	0.1%
Age	65-69			3,147	13.5%	1,896	10.9%	1,251	21.4%	1,389	12.6%	1,096	12.1%	293	14.4%
	70-74			4,590	19.8%	3,106	17.9%	1,484	25.4%	2,120	19.2%	1,683	18.7%	437	21.5%
	75-79			4,696	20.2%	3,446	19.8%	1,250	21.4%	2,223	20.1%	1,815	20.1%	408	20.1%
	80-84			3,514	15.1%	2,691	15.5%	823	14.1%	1,709	15.5%	1,363	15.1%	346	17.0%
	85-89			3,526	15.2%	2,924	16.8%	602	10.3%	1,731	15.7%	1,440	16.0%	291	14.3%
	90+			3,754	16.2%	3,314	19.1%	440	7.5%	1,880	17.0%	1,624	18.0%	256	12.6%
Ethnicity	African			127	0.5%	62	0.4%	65	1.1%	55	0.5%	23	0.3%	32	1.6%
	Any other Asian background			154	0.7%	89	0.5%	65	1.1%	71	0.6%	47	0.5%	24	1.2%
	Any other Black background			65	0.3%	34	0.2%	31	0.5%	35	0.3%	20	0.2%	15	0.7%
	Any other White background			1,084	4.7%	746	4.3%	338	5.8%	490	4.4%	371	4.1%	119	5.9%
	Any other ethnic group			199	0.9%	125	0.7%	74	1.3%	83	0.8%	58	0.6%	25	1.2%
	Any other mixed background			54	0.2%	40	0.2%	14	0.2%	31	0.3%	21	0.2%	10	0.5%

	Bangladeshi or British Bangladeshi	61	0.3%	37	0.2%	24	0.4%	28	0.3%	18	0.2%	10	0.5%
	British, Mixed British	19,165	82.5%	14,768	85.0%	4,397	75.2%	9,137	82.7%	7,647	84.8%	1,490	73.4%
	Caribbean	240	1.0%	94	0.5%	146	2.5%	133	1.2%	54	0.6%	79	3.9%
	Chinese	40	0.2%	15	0.1%	25	0.4%	13	0.1%	6	0.1%	7	0.3%
	Indian or British Indian	323	1.4%	209	1.2%	114	1.9%	160	1.4%	109	1.2%	51	2.5%
	Irish	231	1.0%	185	1.1%	46	0.8%	123	1.1%	99	1.1%	24	1.2%
	Pakistani or British Pakistani	291	1.3%	169	1.0%	122	2.1%	148	1.3%	96	1.1%	52	2.6%
	White and Asian	14	0.1%	9	0.1%	5	0.1%	4	0.0%	3	0.0%	1	0.0%
	White and Black African	10	0.0%	6	0.0%	4	0.1%	5	0.0%	5	0.1%	0	0.0%
	White and Black Caribbean	39	0.2%	21	0.1%	18	0.3%	18	0.2%	9	0.1%	9	0.4%
	Missing	1,130	4.9%	768	4.4%	362	6.2%	518	4.7%	435	4.8%	83	4.1%
NHS Region	East of England	2,549	11.0%	1,997	11.5%	552	9.4%	1,133	10.3%	972	10.8%	161	7.9%
	London	2,390	10.3%	1,704	9.8%	686	11.7%	1,152	10.4%	798	8.8%	354	17.4%
	Midlands	4,749	20.4%	3,554	20.5%	1,195	20.4%	2,451	22.2%	2,012	22.3%	439	21.6%
	North East	4,433	19.1%	3,172	18.3%	1,261	21.6%	2,039	18.4%	1,662	18.4%	377	18.6%
	North West	3,376	14.5%	2,546	14.7%	830	14.2%	1,678	15.2%	1,312	14.5%	366	18.0%
	South East	3,412	14.7%	2,697	15.5%	715	12.2%	1,621	14.7%	1,406	15.6%	215	10.6%
	South West	2,318	10.0%	1,707	9.8%	611	10.4%	978	8.8%	859	9.5%	119	5.9%
IMD Quintiles	1	5,443	23.4%	3,913	22.5%	1,530	26.2%	2,727	24.7%	2,095	23.2%	632	31.1%
	2	4,862	20.9%	3,590	20.7%	1,272	21.7%	2,293	20.7%	1,833	20.3%	460	22.6%
	3	4,575	19.7%	3,441	19.8%	1,134	19.4%	2,118	19.2%	1,739	19.3%	379	18.7%
	4	4,583	19.7%	3,532	20.3%	1,051	18.0%	2,167	19.6%	1,841	20.4%	326	16.1%
	5	3,718	16.0%	2,873	16.5%	845	14.4%	1,723	15.6%	1,496	16.6%	227	11.2%
	Missing	46	0.2%	28	0.2%	18	0.3%	24	0.2%	17	0.2%	7	0.3%
Previously positive	No	20,958	90.2%	15,158	87.2%	5,800	99.1%	9,717	87.9%	7,786	86.3%	1,931	95.1%
	Yes	2,269	9.8%	2,219	12.8%	50	0.9%	1,335	12.1%	1,235	13.7%	100	4.9%
	HSCW**	16	0.1%	12	0.1%	4	0.1%	6	0.1%	5	0.1%	1	0.0%

Vaccine priority groups	CEV***	12,340	53.1%	9,915	57.1%	2,425	41.5%	6,188	56.0%	5,046	55.9%	1,142	56.2%
	At risk****	115	0.5%	69	0.4%	46	0.8%	55	0.5%	47	0.5%	8	0.4%
	Severely immunosuppressed	1,967	8.5%	1,510	8.7%	457	7.8%	1,022	9.2%	795	8.8%	227	11.2%

* ChAdOx1-S primary course
**HSCW: health and social care worker
*** CEV: clinically extremely vulnerable
****At risk is only those under 65 years

Supplementary Figures

Supplementary Figure 1. Flow chart of the data linkage methodology.

Testing data on all positive PCR and LFT tests (Pillar 1 and Pillar 2), and on negative Pillar 2 PCR tests from symptomatic individuals were extracted up to 17 February 2022.

Link to antibody data and extract information for tests which have previously tested positive

Link to NIMS vaccination record on combinations of unique identifiers and extract ethnicity, IMD, at risk flag, CEV flag, severely immunusuppressed flag and vaccine information.

Link to genomics datasets for sequencing and genotyping information

Link to ECDS and SUS hospitalisation records using NHS number, DOB and date of test. ECDS; an emergency admission (not injury) within 14 days of sample date (until day of admission). SUS; Pillar 2 tests- respiratory ICD coded discharge diagnosis (any diagnosis field) where the sample was taken 14 days before and up to 2 days after the day of admission. Pillar 1 tests - respiratory ICD coded discharge diagnosis where the sample was taken 1 days before and up to 2 days after the admission.

Negative tests taken within 7 days of a previous negative test were excluded

Negative tests where symptom onset date was within 10 days of the symptom onset date of a previous negative test were excluded

Negative tests taken within 21 days of a positive test were excluded

For individuals who had more than one negative test, one was selected at random in the study period, and the rest excluded.

Positive and negative tests within 90 days of a previous positive test were dropped. Where participants had later positive tests within 14 days of a positive then preference was given to PCR tests and symptomatic tests.

Cases who received vaccine schedules other than a ChAdOx-S1 primary course followed by a ChAdOxS-1 or BNT162b2 booster were excluded.

Data were restricted to persons aged 40 years and older who had reported symptoms and gave a symptom onset date within the 10 days prior to testing

Data were restricted to Pillar 2 PCR tests between 13 September 2021 to 9 January 2022 for the Delta analysis, and 29 November 2021 to 17 February 2022 for the Omicron analysis.

A small number of positive tests were excluded where sequencing found them to be neither the Delta nor Omicron variant. Some positive tests without sequencing, genotyping or SGTF information were also excluded where

Data were restricted to tests which could be linked to the vaccination record.

For the SUS hospitalisation analysis, data were restricted to those with ARI in the first diagnosis field and where the length of stay was at least two days. Data were restricted to tests up to 2 February 2022 to allow sufficient follow-up time.
