# Supplementary material for: Effectiveness of ChAdOx1-S COVID-19 booster vaccination against the Omicron and Delta variants in England
Source: Nat Commun. 2022 Dec 12;13:7688. doi: 10.1038/s41467-022-35168-7 (PMC9744366; doi:10.1038/s41467-022-35168-7)
Supplement: Supplementary file 3 — Reporting Summary [file 41467_2022_35168_MOESM3_ESM.pdf]

nature portfolio

Corresponding author(s): Freja Kirsebom and Jamie Lopez Bernal

Last updated by author(s): Oct 31, 2022

Reporting Summary

Nature Portfolio wishes to improve the reproducibility of the work that we publish. This form provides structure for consistency and transparency in reporting. For further information on Nature Portfolio policies, see our Editorial Policies and the Editorial Policy Checklist.

Statistics

For all statistical analyses, confirm that the following items are present in the figure legend, table legend, main text, or Methods section.

n/a	Confirmed	
☐	☒	The exact sample size (*n*) for each experimental group/condition, given as a discrete number and unit of measurement
☒	☐	A statement on whether measurements were taken from distinct samples or whether the same sample was measured repeatedly
☒	☐	The statistical test(s) used AND whether they are one- or two-sided<br>*Only common tests should be described solely by name; describe more complex techniques in the Methods section.*
☐	☒	A description of all covariates tested
☒	☐	A description of any assumptions or corrections, such as tests of normality and adjustment for multiple comparisons
☐	☒	A full description of the statistical parameters including central tendency (e.g. means) or other basic estimates (e.g. regression coefficient) AND variation (e.g. standard deviation) or associated estimates of uncertainty (e.g. confidence intervals)
☒	☐	For null hypothesis testing, the test statistic (e.g. *F*, *t*, *r*) with confidence intervals, effect sizes, degrees of freedom and *P* value noted<br>*Give P values as exact values whenever suitable.*
☒	☐	For Bayesian analysis, information on the choice of priors and Markov chain Monte Carlo settings
☒	☐	For hierarchical and complex designs, identification of the appropriate level for tests and full reporting of outcomes
☒	☐	Estimates of effect sizes (e.g. Cohen's *d*, Pearson's *r*), indicating how they were calculated

Our web collection on statistics for biologists contains articles on many of the points above.

Software and code

Policy information about availability of computer code

Data collection	Custom SQL scripts- Microsoft SQL Server Management Studio 18
Data analysis	Custom SQL and Stata scripts- Microsoft SQL Server Management Studio 18 & Stata 15

For manuscripts utilizing custom algorithms or software that are central to the research but not yet described in published literature, software must be made available to editors and reviewers. We strongly encourage code deposition in a community repository (e.g. GitHub). See the Nature Portfolio guidelines for submitting code & software for further information.

Data

Policy information about availability of data

All manuscripts must include a data availability statement. This statement should provide the following information, where applicable:

- Accession codes, unique identifiers, or web links for publicly available datasets
- A description of any restrictions on data availability
- For clinical datasets or third party data, please ensure that the statement adheres to our policy

This work is carried out under Regulation 3 of The Health Service (Control of Patient Information) (Secretary of State for Health, 2002)) (3) using patient identification information without individual patient consent. Data cannot be made publicly available for ethical and legal reasons, i.e. public availability would compromise patient confidentiality as data tables list single counts of individuals rather than aggregated data. Databases used in this study include the National Immunisation Management System (NIMS), Unified Sample Database and the Emergency Care Dataset (ECDS). Aggregated source data (the minimum dataset) are provided with this paper. The raw vaccine effectiveness data are protected and are not available due to data privacy laws.

Human research participants

Policy information about studies involving human research participants and Sex and Gender in Research.

Reporting on sex and gender	Gender was self-reported. Gender was adjusted for in all analyses. Recipients of the AZ booster were more likely to be female. There was no effect of gender on vaccine effectiveness. Overall numbers for all analyses are available in the manuscript.
Population characteristics	Age (5 year bands), sex, index of multiple deprivation (quintile), ethnic group, care home residence status, geographic region (nhs region), period (calendar week of onset), health and social care worker status, clinical risk group status, clinically extremely vulnerable, severely immunosuppressed, and previously testing positive. Full details of the population characteristics are included in the manuscript in Supplementary Tables 3 and 6.
Recruitment	Observational study using routinely collected data as part of the UK COVID-19 testing and vaccination programme
Ethics oversight	UKHSA Research Ethics and Governance Group Statement: Surveillance of COVID-19 testing and vaccination is undertaken under Regulation 3 of The Health Service (Control of Patient Information) Regulations 2002 to collect confidential patient information (http://www.legislation.gov.uk/uksi/2002/1438/regulation/3/made) under Sections 3(i) (a) to (c), 3(i)(d) (i) and (ii) and 3(3). The study protocol was subject to an internal review by the PHE Research Ethics and Governance Group and was found to be fully compliant with all regulatory requirements. As no regulatory issues were identified, and ethical review is not a requirement for this type of work, it was decided that a full ethical review would not be necessary.<br>All necessary patient/participant consent has been obtained and the appropriate institutional forms have been archived.

Note that full information on the approval of the study protocol must also be provided in the manuscript.

Field-specific reporting

Please select the one below that is the best fit for your research. If you are not sure, read the appropriate sections before making your selection.

☐ Life sciences ☒ Behavioural & social sciences ☐ Ecological, evolutionary & environmental sciences

For a reference copy of the document with all sections, see nature.com/documents/nr-reporting-summary-flat.pdf

Behavioural & social sciences study design

All studies must disclose on these points even when the disclosure is negative.

Study description	Test negative case control design that compares the vaccination status in COVID-19 positive and negative cases using quantitative data.
Research sample	Not a sample - this study included the whole English resident population, and is by definition representative. The relevant demographic details are described in full in Supplementary Tables 3 and 6.
Sampling strategy	There was no sampling strategy since this dataset included the whole English resident population (not a sample).
Data collection	Routinely collected data: National COVID-19 vaccine register and national COVID-19 testing data. These data are used for clinical management and disease surveillance purposes. Data were extracted using custom SQL scripts. No instruments were used to obtain data. Blinding is not applicable here since the data are observational data used for an epidemiological study.
Timing	Data on AZ booster vaccine recipients were extracted on 14 March 2022. The booster vaccination programme commenced in

	September 2021 as recommended by the JCVI; this was when the first individuals received a booster vaccine. VE analysis: The Delta analysis was restricted from 13 September 2021 to 9 January 2022. The Omicron analysis was restricted from 29 November to 17 February 2022.
Data exclusions	Any negative tests taken within 7 days of a previous negative test, and any negative tests where symptom onset date was within the 10 days or a previous symptoms onset date for a negative test were dropped as these likely represent the same episode. Negative tests taken within 21 days of a subsequent positive test were also excluded as chances are high that these are false negatives. Positive and negative tests within 90 days of a previous positive test were also excluded; however, where participants had later positive tests within 14 days of a positive then preference was given to PCR tests and symptomatic tests. For individuals who had more than one negative test, one was selected at random in the study period. Data were restricted to persons who had reported symptoms and gave a symptom onset date within the 10 days before testing to account for reduced PCR sensitivity beyond this period in an infection event.
Non-participation	No participants were involved in the study
Randomization	No randomization was needed as this is an observational study

Reporting for specific materials, systems and methods

We require information from authors about some types of materials, experimental systems and methods used in many studies. Here, indicate whether each material, system or method listed is relevant to your study. If you are not sure if a list item applies to your research, read the appropriate section before selecting a response.

Materials & experimental systems

n/a	Involved in the study
☒	☐ Antibodies
☒	☐ Eukaryotic cell lines
☒	☐ Palaeontology and archaeology
☒	☐ Animals and other organisms
☒	☐ Clinical data
☒	☐ Dual use research of concern

Methods

n/a	Involved in the study
☒	☐ ChIP-seq
☒	☐ Flow cytometry
☒	☐ MRI-based neuroimaging
